# Comparison of the Effects of Haloperidol, Metoclopramide, Dexmedetomidine and Ginger on Postoperative Nausea and Vomiting After Laparoscopic Cholecystectomy

**DOI:** 10.25122/jml-2019-0070

**Published:** 2020

**Authors:** Amir Reza Naemi, Vahid Kashanitabar, Alireza Kamali, Ashkan Shiva

**Affiliations:** 1. Department of Surgery, Arak University of Medical Sciences, Arak, Iran; 2. Department of Anesthesiology and Critical Care, Arak University of Medical Sciences, Arak, Iran

**Keywords:** Dexmodetomidine, Haloperidol, Plasil, Zinger extract, nausea, vomiting, laparoscopy

## Abstract

Nausea is a mental sensation of unease and discomfort before vomiting. Vomiting refers to the return of the contents of the upper gastrointestinal tract to the mouth caused by contractions of chest and abdomen muscles.

Postoperative nausea and vomiting is an unpleasant experience with high treatment costs. Therefore, this study aimed to compare the effects of haloperidol, metoclopramide, dexmedetomidine, and ginger on postoperative nausea and vomiting after laparoscopy.

This double-blind clinical trial was performed on all laparoscopy candidates at Valiasr hospital, Arak, Iran. The patients were randomly divided into four groups (haloperidol, metoclopramide, dexmedetomidine and ginger), and all patients underwent general anesthesia using fentanyl, midazolam, atracurium, and propofol. After intubation, tube fixation, and stable hemodynamic conditions, the patients received four ginger capsules with a hint of lemon. A group of patients received 25 μg of dexmedetomidine. In the Plasil group, 10 mg of metoclopramide was given 30 minutes before the completion of surgery. In addition, 0.5 cc of haloperidol (5 mg) was administered to a group of patients. Heart rate, blood pressure, and oxygen saturation were recorded from the beginning of surgery, every 15 minutes until the end of the surgery. Furthermore, the occurrence of nausea and vomiting was recorded during recovery, 2 and 4 hours after surgery. Data were then analyzed using the SPSS software v.23.

Eighty-eight patients were enrolled in the study. The youngest and the oldest were 30 years and 70 years old, respectively, and the mean age was 48.02 ± 9.31 years. Moreover, the number of women in the four groups was higher than that of men. Blood pressure in the dexmedetomidine group was lower than the other four groups (P <0.05). The lowest heart rate was observed in the haloperidol group, while the highest heart rate was seen in the plasil group (P <0.05). The occurrence of vomiting and nausea was not significantly different between the four groups (P <0.05).

Our results showed no significant difference in postoperative nausea and vomiting between the four drugs. Due to the hemodynamic changes induced by each drug, it is best to use these drugs based on the patient’s condition. Ginger is also a herbal remedy that has fewer side effects, and this drug can be a good option for patients when there is no contraindication.

## Introduction

Nausea is a mental sensation of unease and discomfort before vomiting. Vomiting refers to the return of the upper gastrointestinal tract contents to the mouth caused by contractions of chest and abdomen muscles [1]. Postoperative nausea and vomiting occur in 20 to 30% of patients, although in some conditions, this rate increases to 70%, and both are the second most common complication after surgery [2, 3]. This postoperative complication is one of the patient’s dissatisfactions in the postoperative period [4, 5].

Factors affecting postoperative nausea and vomiting include age, sex, obesity, BMI> 30 kg/ m2, and anxiety. Postoperative nausea and vomiting triggers include gastroparesis after anesthesia, excessive hunger, hypoglycemia, and vasoactive drugs [6, 7]. Vomiting during the operation is associated with a risk of visceral injury, prolonged operation, and aspiration risk, leading to stress and disruption of the surgical procedure [8].

Research focuses on methods and drugs with a low side-effect risk in reducing postoperative nausea and vomiting. The ideal medication should have the proper effect and the least side effects.

The laparoscopic technique used in cholecystectomy is a procedure that commonly leads to complications such as nausea and vomiting. The advantages of this method are the reduced hospitalization duration and reduced postoperative complications rate [9, 10]. Laparoscopic surgery is a type of surgical procedure in which a laparoscope is inserted into a small incision to visualize abdominal organs. Laparoscopy is used to find conditions such as cysts, adhesions, infections, and fibroids in the uterine masses. Also, a part of a certain tissue can be removed, and a biopsy can be obtained using a laparoscope. This method has recently been used to diagnose and treat many diseases. During laparoscopy, the pneumoperitoneum can produce vagal stimulation, leading to nausea and vomiting [11]. Currently, various drugs are used to prevent and treat nausea and vomiting after surgery, including haloperidol, metoclopramide, dexmedetomidine, and ginger extract.

Butyrophenone haloperidol can produce anti-nausea and vomiting effects by inhibiting dopamine D2 receptors (D2R) in the central nervous system [12]. Dexmedetomidine is a selective alpha-2 adrenergic receptor agonist with sedative, anti-anxiety, and analgesic effects [11].

Metoclopramide is another anti-vomiting drug with dopaminergic effects [13]. Ginger-based anti-nausea and anti-vomiting compounds include gingerol and Shuga. Unlike other anti-nausea drugs that affect the central nervous system, ginger has local anesthetic effects on the stomach and intestines due to its anticholinergic and anti-histaminic properties [14]. So far, no comparison has been made on the effects of these drugs for the prevention of nausea and vomiting. On the other hand, the use of dexmedetomidine has other beneficial effects, other than its anti-nausea and anti-vomiting effects, and can be a good alternative to other drugs.

However, the use of herbal compounds such as ginger has had a lot of supporters over the past few years due to the lack of complications that are usually seen in chemical drugs.

Therefore, the study was performed to compare the effects of haloperidol, metoclopramide, dexmedetomidine, and ginger extract on nausea and vomiting after laparoscopy.

## Material and Methods

This double-blind clinical trial was conducted on all laparoscopy candidates at Valiasr hospital, Arak, Iran. The participants who entered the study were randomly divided into four groups (haloperidol, metoclopramide, dexmedetomidine, and ginger extract).

Inclusion criteria:

1. Patients signing an informed consent
2. ASA I and II patients
3. Lack of history of psychiatric and psychotic illnesses
4. Lack of Parkinson’s disease, motor illness or history of chemotherapy
5. Patients aged 18-60 years
6. Surgery lasting between 90 to 150 minutes

Exclusion criteria:

1. Unsatisfied patients
2. ASA III and ≥ IV patients
3. Patients outside the age range of 35-60 years
4. Duration of surgery for more than 150 minutes
5. Parkinson’s disease
6. Psychological and psychological diseases
7. History of chemotherapy

At first, informed consent was obtained from all patients. After the anesthetic confirmation, the patients entered the operating room and were monitored for peripheral oxygen saturation, heart rate, blood pressure, noninvasive blood pressure, and body temperature. Then, all patients received 3-5 ml/kg of crystalloid fluid as compensatory intravascular volume expansion (CVE), fentanyl (2 mg/kg), midazolam (0.3 to 0.5 mg/kg), atracurium (0.0 to 7.0 mg/kg), and propofol (2-3 mg/kg) for general anesthesia.

After intubation, tube fixation, and stable hemodynamic conditions, the patients received four ginger capsules with a hint of lemon. In addition, the other group received 25 μg of dexmedetomidine. In the Plasil group, 10 mg of metoclopramide was given 30 minutes before the completion of surgery. In the next group, patients received 0.5 cc of haloperidol (5 mg) and Table 1 shows the vomiting score that was considered.

**Table 1:** Vomiting score.

**Vomiting Score**	**Definition**
**0**	The patient reports no vomiting or similar symptoms
**1**	The patient has manageable retch
**2**	The patient has moderate retch and vomiting 1 to 2 times, which is manageable
**3**	The patient has frequent retch and frequent vomiting, which is difficult to control
**4**	The patient has uncontrollable, recurrent vomiting

The incidence of nausea was measured based on the presence or absence of nausea in patients in recovery and 2 and 4 hours after surgery.

Heart rate, blood pressure and oxygen saturation were recorded from the beginning of surgery, every 15 minutes, until the end of the surgery. The occurrence of nausea and vomiting was also recorded during recovery, 2, and 4 hours after surgery.

The sample size was calculated as follows:


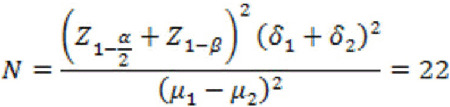


Taken together, the patients were divided into four groups of 22 participants each.

### Data analysis

Data were evaluated using the SPSS software v.23. ANOVA tests were used to analyze parametric and nonparametric data among groups. Furthermore, a Chi-square test was used.

1. An introduction letter was obtained from the university officials
2. An introduction letter was received from the officials of selected centers
3. The purpose of the study was described for all participants, and written consent was then obtained.

## Results

A total of 88 patients were enrolled in 4 groups and then evaluated for mean and age deviation. In the four groups, the youngest and the oldest were 30 years and 70 years, respectively. The mean age was 48.02 ± 9.31 years. There were no significant differences between the four groups in terms of surgical duration (P = 679.0) and age (P = 323.0). In the present study, six men (27.27%) and 16 women (72.72%) were entered into the Plasil group, followed by two men (9.9%) and 20 women (90.90%) in the dexmedetomidine group, 10 men (45.45%) and 12 women (54.54 %) in haloperidol group and 2 men (09.9%) and 20 women (90.90%) in the ginger group. According to the obtained data, there was a significant difference between all groups in terms of gender (P = 0.10), indicating a higher number of women.

The mean and standard deviation of blood pressure are shown in Table 2. Blood pressure at baseline did not show a significant difference in the four groups (P = 0.896), indicating that the baseline blood pressure was nearly equal in the four groups. However, blood pressure was statistically different between the groups (P <0.05) postoperatively. At all times, blood pressure in the dexmedetomidine group was lower compared to the other groups.

**Table 2:** Mean and standard deviation of blood pressure.

**Group** **Blood pressure**	**Plasil** **Mean ± SD**	**Dexmedmotidine** **Mean ± SD**	**Hallopridol** **Mean ± SD**	**Ginger** **Mean ± SD**	**p-value**
**First**	14.4 ± 81.77	84.3 ± 95.77	09.4 ± 68.77	16.4 ± 63.78	0.896
**15 minutes after surgery**	26.5 ± 09.89	77.4 ± 18.76	15.5 ± 59.75	68.3 ± 59.77	0.0001
**30 minutes after surgery**	36.1 ± 81.75	52.4 ± 09.73	82.4 ± 68.72	66.5 ± 63.75	0.032
**45 minutes after surgery**	76.5 ± 45.74	67.3 ± 90.70	71.4 ± 63.71	55.5 ± 72.74	0.024
**60 minutes after surgery**	45.4 ± 22.74	48.4 ± 36.69	33.4 ± 13.69	96.3 ± 27.74	0.0001
**75 minutes after surgery**	27.5 ± 54.75	40.5 ± 86.69	36.5 ± 40.70	40.5 ± 86.74	0.10001
**90 minutes after surgery**	74.3 ± 18.75	89.4 ± 36.70	48.4 ± 00.70	08.4 ± 86.73	0.0001
**105 minutes after surgery**	88.5 ± 18.74	45.5 ± 54.70	30.5 ± 68.70	23.4 ± 50.76	0.0001

As shown in Table 3, there was no significant difference in oxygen saturation at all times after surgery among the four groups (P> 0.05), indicating that the oxygen saturation percentage at all times was almost the same among four groups.

**Table 3:** Average and standard deviation of oxygen saturation.

**Group** **Oxygen saturation**	**Plasil** **Mean ± SD**	**Dexmedmotidine** **Mean ± SD**	**Hallopridol** **Mean ± SD**	**Ginger** **Mean ± SD**	**p-value**
**First**	657.0 ± 63.99	716.0 ± 68.99	646.0 ± 68.99	666.0 ± 59.99	0.964
**15 minutes after surgery**	801.0 ± 50.99	789.0 ± 36.99	922.0 ± 22.99	827.0 ± 27.99	0.715
**30 minutes after surgery**	00.00 ± 00.100	351.0 ± 86.99	908.0 ± 59.99	646.0 ± 68.99	0.098
**45 minutes after surgery**	651.1 ± 18.98	053.1 ± 40.98	245.1 ± 13.98	390.1 ± 136.98	0.893
**60 minutes after surgery**	351.1 ± 27.98	326.1 ± 045.98	098.1 ± 59.98	345.1 ± 00.98	0.409
**75 minutes after surgery**	306.1 ± 22.98	230.1 ± 09.98	181.1 ± 40.98	540.1 ± 09.98	0.835
**90 minutes after surgery**	192.1 ± 22.98	882.0 ± 72.98	125.1 ± 13.98	056.1 ± 54.98	0.233
**105 minutes after surgery**	386.1 ± 27.98	129.1 ± 31.98	477.1 ± 22.98	42.1 ± 86.97	0.674

The mean and standard deviation of vomiting are shown in Table 4. All groups had no superiority in terms of their effect on vomiting reduction during recovery, 2 and 4 hours after surgery (P> 0.05).

**Table 4:** Mean and standard deviation of vomiting.

**Group** **Vomiting**	**Plasil** **Mean ± SD**	**Dexmedmotidine** **Mean ± SD**	**Hallopridol** **Mean ± SD**	**Ginger** **Mean ± SD**	**p-value**
**Recovery**	670.0 ± 454.0	455.0 ± 272.0	800.0 ± 545.0	657.0 ± 363.0	0.555
2 hours after surgery	631.0 ± 272.0	492.0 ± 363.0	134.0 ± 631.0	394.0 ± 181.0	0.749
4 hours after surgery	294.0 ± 090.0	294.0 ± 090.0	394.0 ± 181.0	294.0 ± 090.0	0.272

There was no significant difference in terms of nausea occurrence during recovery (P = 0.84), 2 hours (P = 0.861), and 4 hours after surgery (P = 0.141), indicating that these four groups had no superiority over each other to reduce nausea (Table 5).

**Table 5:** Frequency and incidence of nausea.

**Group** **Nausea**		**Plasil** **Number (percent)**	**Dexmedmotidine** **Number (percent)**	**Hallopridol** **Number (percent)**	**Ginger** **Number (percent)**	**p-value**
**Recovery**	**yes**	8 (36.36)	6 (27.27)	8 (36.36)	6 (27.27)	0.840
	**no**	14 (63.63)	16 (72.72)	14 (63.63)	16 (72.72)	
**2 hours after surgery**	**yes**	8 (36.36)	10 (45.45)	10 (45.45)	8 (36.36)	0.861
	**no**	14 (63.63)	12 (54.54)	12 (54.54)	14 (63.63)	
**4 hours after surgery**	**yes**	6 (27.27)	10 (45.45)	10 (45.45)	8 (36.36)	0.141
	**no**	16 (72.72)	12 (54.54)	12 (54.54)	14 (63.63)	

## Discussion

Postoperative nausea and vomiting is a highly stressful complication. Drug and non-drug therapies are being used to reduce these complications before and after surgery, and even during recovery and admission [15].

Therefore, we compared the effect of haloperidol, metoclopramide, dexmedetomidine, and ginger extract on postoperative nausea and vomiting. According to the results, the number of women in this study was higher than that of men, which is consistent with the high prevalence of laparoscopic procedures in women. In this study, blood pressure in the dexmedetomidine group was lower than it was in the other groups.

Shenhui et al. assessed the effect of dexmedetomidine on postoperative nausea and vomiting, where dexmedetomidine was capable of reducing nausea and vomiting and as well as side effects such as bradycardia and hypotension [16]. These findings were in agreement with our results.

Another study by Geng et al. aimed at studying the effect of dexmedetomidine on nausea and vomiting following gynecological laparoscopic surgery. They indicated that dexmedetomidine was capable of reducing postoperative nausea, but not vomiting in the 24-hour period after surgery [17]. Our study also indicated a reduction in nausea and vomiting with the use of dexmedetomidine.

In the present study, the haloperidol group exhibited lower blood pressure than the metoclopramide and ginger groups. The lowest heart rate was observed in the haloperidol group.

Yahyavi and Nazari conducted a study in 2004 with the aim of evaluating the effect of haloperidol on the prevention of nausea and vomiting following women’s surgery. Using a dose of haloperidol before the induction of anesthesia was able to reduce nausea and vomiting and delayed their occurrence. It was also effective in reducing the episode of vomiting and reducing the need for postoperative therapeutic interventions [18]. The results of the mentioned study were consistent with our findings because haloperidol was capable of reducing nausea and vomiting and decreasing blood pressure and heart rate in the present study.

The incidence of vomiting and nausea was not statistically significant at all times in the four groups (P <0.05). Our results did not show a difference in nausea and vomiting between the four drugs after surgery. Due to the hemodynamic changes induced by each drug, it is advisable to use these drugs based on the patient’s condition.

The lowest level of hemodynamic changes was observed in the ginger group. This drug is also an herbal remedy and has fewer side effects and can be a good option for patients when there is no contraindication.

In a review by Stack et al. from 2016, the effect of consuming various kinds of ginger products on the reduction of nausea and vomiting during pregnancy had been investigated. They indicated that ginger, its active ingredient along with other compounds of this plant, has numerous pharmacological effects. The results indicated that various ginger forms, such as a biscuit or a capsule, reduce nausea and vomiting in pregnant women [19]. Their results were consistent with our study.

Another study assessed the effects of ginger capsules on nausea and vomiting during pregnancy, and ginger was an effective herbal remedy for decreasing pregnancy nausea and vomiting in comparison with the placebo group [20]. In our study, ginger also reduced nausea and vomiting without any changes in hemodynamic parameters.

## Conclusion

There was no significant difference in the occurrence of nausea and vomiting in each of the four groups (haloperidol, metoclopramide, dexmedetomidine and ginger) after surgery. Due to the hemodynamic changes induced by each drug, it is best to use these drugs based on the patient’s condition. Ginger is also a herbal remedy that has fewer side effects and could be a good option for patients when there is no contraindication.

## Conflict of Interest

The authors declare that there is no conflict of interest.
